# Reductions of copper ion-mediated low-density lipoprotein (LDL) oxidations of trypsin inhibitors, the sweet potato root major proteins, and LDL binding capacities

**DOI:** 10.1186/s40529-020-00303-4

**Published:** 2020-09-24

**Authors:** Yeh-Lin Lu, Chia-Jung Lee, Shyr-Yi Lin, Wen-Chi Hou

**Affiliations:** 1 School of Pharmacy, College of Pharmacy, Taipei Medical University, Taipei, 110 Taiwan; 2 Ph.D. Program in Clinical Drug Development of Herbal Medicine, College of Pharmacy, Taipei Medical University, Taipei, Taiwan; 3 Division of Gastroenterology, Department of Internal Medicine, Wan Fang Hospital, Taipei Medical University, Taipei, Taiwan; 4 Department of General Medicine, School of Medicine, College of Medicine, Taipei Medical University, Taipei, Taiwan; 5 Graduate Institute of Pharmacognosy, Taipei Medical University, No. 250, Wu-Hsing Street, Taipei, 110 Taiwan

**Keywords:** Low-density lipoprotein (LDL), Thiobarbituric-acid-reactive-substance (TBARS), Sweet potato trypsin inhibitors (SPTIs)

## Abstract

**Background:**

The root major proteins of sweet potato trypsin inhibitors (SPTIs) or named sporamin, estimated for 60 to 80% water-soluble proteins, exhibited many biological activities. The human low-density lipoprotein (LDL) showed to form in vivo complex with endogenous oxidized alpha-1-antitrypsin. Little is known concerning the interactions between SPTIs and LDL in vitro.

**Results:**

The thiobarbituric-acid-reactive-substance (TBARS) assays were used to monitor 0.1 mM Cu^2+^-mediated low-density lipoprotein (LDL) oxidations during 24-h reactions with or without SPTIs additions. The protein stains in native PAGE gels were used to identify the bindings between native or reduced forms of SPTIs or soybean TIs and LDL, or oxidized LDL (oxLDL). It was found that the SPTIs additions showed to reduce LDL oxidations in the first 6-h and then gradually decreased the capacities of anti-LDL oxidations. The protein stains in native PAGE gels showed more intense LDL bands in the presence of SPTIs, and 0.5-h and 1-h reached the highest one. The SPTIs also bound to the oxLDL, and low pH condition (pH 2.0) might break the interactions revealed by HPLC. The LDL or oxLDL adsorbed onto self-prepared SPTIs-affinity column and some components were eluted by 0.2 M KCl (pH 2.0). The native or reduced SPTIs or soybean TIs showed different binding capacities toward LDL and oxLDL in vitro.

**Conclusion:**

The SPTIs might be useful in developing functional foods as antioxidant and nutrient supplements, and the physiological roles of SPTIs-LDL and SPTIs-oxLDL complex in vivo will investigate further using animal models.

## Background

Sweet potato (SP) (*Ipomoea batatas* [L.] Lam) is ranked the 3rd productions after potato and cassava among the five major tuberous crops (potato, cassava, SP, yam, and taro) in the world, which account for about 99% total productions of tuberous crops worldwide (FAO 1999; Shewry 2003). The nutritional values of carbohydrates in tuberous crops are mainly for dietary energy supplies; and the approximate protein contents are 3–6%, 1–10%, and 1–2% of dry weight, respectively, for potato, SP, and cassava (Shewry 2003), or varies among 1–2 g per 100-g edible portion (FAO 1990). SP roots also contain higher dietary fibers, beta-carotene, and vitamins (especial for thiamine, ascorbic acid and folic acid) among major tuberous crops, which are used as staple foods or foods, animal feeds, and industrial starch processing (Mohanraj and Sivasankar 2014; Heuzé et al. 2015). Except roots, SP leaves contains phenolic compounds, such as lutein, quercetin, caffeoylquinic acid derivatives, and chlorogenic acid, which provide protection against aging, cancer, allergies, diabetes, hypertension, and cardiovascular diseases (Mohanraj and Sivasankar 2014).

Sohonie and Bhandarker (1954) described the trypsin inhibitory activities in SP roots, which was the first report in non-leguminous plants. Lin and Chen (1980) reported that the trypsin inhibitory activities of water extracts from 53 varieties of SP roots in Taiwan exhibited heat-resistant and heat-labile properties, which the trypsin inhibitory activities might be as useful taxonomic traits in SP varieties (Lin 1988). Maeshima et al. (1985) purified and named sporamin as the major storage proteins in SP roots. Later, the overexpressed sporamin in *E. coli* showed trypsin inhibitory activity (Yeh et al. 1997; Senthilkumar and Yeh 2012). The purified SPTIs have been reported to exhibit in vitro biological activities, such as dehydroascorbate eductase and momodehydroascorbate reductase activities (Hou and Lin 1997a), antioxidant activities (Hou et al. 2001, 2005), glutathione peroxidase-like activities (Hou et al. 2004), angiotensin converting enzyme inhibitory activities (Huang et al. 2006), thioltransferase-like and glutathione S-transferase-like activities (Huang et al. 2009). Huang et al. (2008) reported that BALB/c mice fed normal diet concurrent with SPTIs (100 mg/kg) by gavaging once every 2 days for 35 days. They found that the SPTIs interventions showed significantly to increase plasma antioxidant activities by ABTS method and reduce hepatic malondialdehyde (MDA) and plasma triglyceride levels compared to the control (*P* < 0.05 or 0.01), however, no significant change of plasma low-density-lipoproteins (LDL)-cholesterol (C) level was reported. Ishiguro et al. (2016) used three heat-stable proteases at pH 8.5, 65 °C for 16 h to treat SP proteins in order to get SP peptides (SPP). The BALB/c mice fed high-fat diet containing 0.5% or 5% SPP (W/W of high-fat diet) for 28 days significantly showed to reduce body weight, epididymal fats and mesenteric fats, plasma triglyceride, and LDL-C compared to those in the control fed high-fat diet only.

The oxidized LDL (oxLDL) is an important factor in atherosclerotic pathogenesis (Kawamura et al. 1994). Therefore, Cu^2+^-mediated LDL oxidation was a general method in vitro to investigate effects of natural compounds or extracts on delaying LDL oxidations, which the associated mechanisms might involve Cu^2+^-mediated destructions of Trp residues in apoB portion of LDL to generate Trp radicals, which participated in advance to unsaturated lipid oxidations (Gießauf et al. (1995); Schnitzer et al. 1997; Knott et al. 2002; Nakano et al. 2004). Wu et al. (2009) reported that the tested phenolic compounds, such as luteolin, naringenin, kaempferol, quercetin, showed to bind LDL and enhance in vitro protections against Cu^2+^-mediated LDL oxidations detected by thiobarbituric-acid-reactive-substance (TBARS) assays. Wu et al. (2009) reported that the tested phenolic compounds, such as luteolin, naringenin, kaempferol, quercetin, showed to bind LDL and enhance in vitro protections against Cu^2+^-mediated LDL oxidations detected by thiobarbituric-acid-reactive-substance (TBARS) assays. Mashiba et al. (2001) reported that human oxidized (inactivated) plasma α_1_-antitrypsin (oxidized AT) could form complex with LDL and co-eluted in the gel permeation chromatography, and this complex might play anti-atherosclerotic effects in vivo (Kotani et al. 2010). Therefore, in this study, the TBARS assays were used to monitor 0.1 mM Cu^2+^-mediated LDL oxidations during 24-h reactions with or without SPTIs additions. The protein stains in native PAGE gels were used to identify the bindings between native or reduced forms of SPTIs and LDL, oxLDL, and commercial soybean TIs were used for comparisons.

## Methods

### Materials

Copper (II) sulfate, 2-thiobarbituric acid, and trypsin (TPCK-treated, 40 U/mg) were purchased from E. Merck Inc. (Darmstadt, Germany). Soybean TI was purchased from Roche Applied Science (Mannheim, Germany). Dithiothreitol, EDTA.2Na, electrophoretic reagents, human LDL, and other chemicals and reagents were purchased from Sigma Chemical Co. (St. Louis, MO, USA). Thiolyte^®^ (monobromobimane reagent, mBBr, cat. 596105) was from Millipore Co. (MA, USA). The prestained protein marker for SDS-PAGE was from Bioman Sci. Co. LTD (Taiwan). The disposable PD-10 desalting column and CNBr-activated Sepharose 4B resins were purchased from Amersham Biosciences (Uppsala, Sweden).

### Plant materials and TI purification

Fresh roots of SP (*Ipomoea batatas* (L.) Lam ‘Tainong 57’) were purchased from a supermarket. Extraction and purification processes were according to previous methods (Hou and Lin 1997a, b; Hou et al. 2001, 2004, 2005). The SP crude extracts were loaded to an affinity column of self-prepared trypsin-Sepharose 4B resins, and the adsorbed TIs were eluted by changing pH value with 0.2 M KCl buffer (pH 2.0), the absorbed SPTIs proteins were collected, adjusted to pH 7.5, dialyzed against distilled water, and then lyophilized for further uses.

### Protections against Cu^2+^-mediated LDL oxidation by SPTIs

The capacity of purified SPTIs to prevent Cu^2+^-mediated human LDL oxidation was measured by TBARS assay using absorbance at 532 nm following the previous method (Hou et al. 2005). After dialysis against 10 mM phosphate buffer (pH 7.4) overnight to remove EDTA, the total 100 μl solution contained 20 μl of dialyzed LDL (2 mg/ml), 40 μl of SPTIs (1 mg/ml), and tenfold-diluted PBS containing 10 μl of 1 mM copper (II) sulfate at 37 °C for 24 h. The tenfold-diluted PBS instead of SPTIs solution was used in the control. During reaction time interval of 0.5-, 1- 2-, 3-, 4-, 6-, and 24-h, the Cu^2+-^mediated reaction was stopped by adding 10 μl of 1 mM EDTA solution, and then for TBARS assay. The EDTA was added before copper (II) sulfate in the LDL/SPTIs mixture was used for the zero time assay.

### SPTIs bound to LDL during Cu^2+^-mediated LDL oxidation

The dialyzed LDL (10 μl, 5.8 μg) mixed with SPTIs (20 μl, 20 μg), copper (II) sulfate (5 μl, 100 μM in the final concentration), and tenfold-diluted PBS (15 μl) were reacted at 37 °C for 0, 0.5, 1, 2, 4, 8, and 24 h. The dialyzed LDL mixed with copper (II) sulfate performed in the parallel experiment. The reaction was stopped by adding 5 μl of 1 mM EDTA solution. The SPTIs bound LDL during Cu^2+^-mediated LDL oxidation was carried out on a discontinuous Tris–glycine native polyacrylamide gel electrophoresis (PAGE) gel system, one 4% stacking gel and two layers of separation gel of 5% gel (4 cm) and 12.5% gel (2.5 cm). After electrophoresis, the native gel was fixed with 12.5% trichloroacetic acid for at least 30 min, and stained by Comassie brilliant blue R-250 for proteins. The images in the selected square frame of stained proteins were quantified by a Syngene G:bBOX imaging system (Syngene, UK).

### SPTIs bound to oxLDL revealed by native PAGE gels and HPLC

The dialyzed LDL was reacted with copper (II) sulfate at 37 °C for 24 h, and the reaction was terminated by adding EDTA solution (the final concentration of 100 μM) as the oxLDL. The solution was stored at 4 °C for further uses. The different amounts of SPTIs (1, 5, 10, and 15 μg) was mixed with oxLDL (about 10 μg) in tenfold-diluted PBS at 37 °C for 30 min. The SPTIs bound oxLDL was carried out on a discontinuous Tris–glycine native PAGE gel system, one 4% stacking gel and two layers of separation gel of 4% gel (4 cm) and 20% gel (2.5 cm). After electrophoresis, the gel was placed in 12.5% trichloroacetic acid solution for 30 min, and stained proteins by Comassie brilliant blue R-250. The images in the selected square frame of stained proteins were quantified by a Syngene G:bBOX imaging system (Syngene, UK). For HPLC analysis, the total 300 μl mixture contained SPTIs (120 μg) and oxLDL (120 μg) in tenfold-diluted PBS for 1 h. The SPTIs/oxLDL solution was rapidly mixed either with equal volume of distilled water or glycine–HCl buffer (pH 2.0), and the molecular size shift was analyzed by a TSKgel G3000PW_XL_ (7.8 × 300 mm) gel filtration column in the Breeze HPLC system. The SPTIs alone in glycine–HCl buffer (pH 2.0) was used for comparisons. The Breeze HPLC system (Waters Co., MA, USA) was equipped with pump (Waters 1525) and UV detector (Water 2487). The 10 mM Tris–HCl buffer (pH 7.9) containing 0.1 M NaCl was used as the mobile phase with flow rate of 0.5 ml/min. Each 20 μl sample was injected and the absorbance at 280 nm was monitored.

### LDL or oxLDL bound onto self-prepared SPTIs-Sepharose 4B affinity column

The preparation of SPTIs-Sepharose 4B affinity column was according to the previous method (Hou and Lin 2002) using CNBr-activated Sepharose 4B resins. About 300 μg LDL or oxLDL in tenfold-diluted PBS were loaded onto the SPTIs-Sepharose 4B affinity column. After being washed with tenfold-diluted PBS for 15 fractions and 10 mM Tris–HCl buffer (pH 7.9) containing 100 mM NaCl for another 15 fractions, the bound ones were eluted by 0.2 M KCl (pH 2.0). Each fraction was monitored the absorbance at 280 nm. The flow rate was 30 ml/h and each tube contained 5.0 ml. The unbound fractions in tenfold-diluted PBS washing were designed as fraction 1, and the bound fractions eluted by 0.2 M KCl buffer (pH 2.0) were saved, adjusted to 7.4, as fraction 2. Each fraction was concentrated with centriprep 10 to small volumes to perform native gel electrophoresis.

### Reductions and free thiol-labeling of SPTIs and soybean TIs, activity stains in SDS-PAGE gels, and LDL or oxLDL bindings

The reductions of SPTIs or soybean TIs by dithiothreitol (DTT) were freshly prepared according to the previous methods (Trümper et al. 1994). The equal volume of purified SPTIs and soybean TI at 2 mg/ml were mixed with 20 mM DTT in tenfold-diluted PBS for 2 h, and the excess of DTT was removed by the disposable PD-10 column balanced with distilled water. The free thiol-labeling in native or reduced SPTIs or commercial soybean TIs were achieved by thiolyte^®^ fluorescent dyes following the previous methods (Kobrehel et al. 1991; Hou and Lin 1997a), and then performed electrophoresis. After electrophoresis, the gel was stained for proteins by Comassie brilliant blue R-250; for TI activity stains, the SDS was removal by 25% isopropanol in 10 mM Tris buffer (pH 7.9), and then the gel was placed in trypsin solution at 37 °C for 30 min for gentle shakings. The gel was stained in the 80 ml substrate-dye solution containing 20 mg *N*-acetyl-phenylalanine β-naphthyl ester and 40 mg tetrazotized *O*-dianisidine under light protections at 37 °C for 30 min (Hou and Lin 1997a, b), and the blank zones against deep purple background showed TI positions. For comparing different binding capacities of SPTIs, reduced SPTIs, soybean TIs, and reduced soybean TIs toward LDL and oxLDL, the native or reduced TIs (10 μg) and LDL or oxLDL (10 μg) in tenfold-diluted PBS were mixed for 1 h, and then were carried out on a discontinuous Tris–glycine native PAGE gel system as above-mentioned.

### Statistical analysis

Data were expressed as mean ± SD. Multiple group comparisons were performed using one-way analysis of variance (ANOVA) and the post hoc Tukey’s test, and the different uppercase alphabet in each treatment or lowercase alphabet in each treatment were considered significantly different (*P *< 0.05). The student’s t-test was used for two group comparisons of binding capacities between LDL and (LDL + native or reduced forms of SPTIs or soybean TIs), or oxLDL and (oxLDL + native or reduced forms of SPTIs or soybean TIs). A difference was considered statistically significant when *P* < 0.05 (*) or *P* < 0.01 (**) or *P* < 0.001 (***). The GraphPad Prism 6 (San Diego, CA) was used for statistical analysis.

## Results

### Effects of SPTIs on Cu^2+^-mediated LDL oxidation and SPTIs bound to oxLDL

**Fig. 1:**
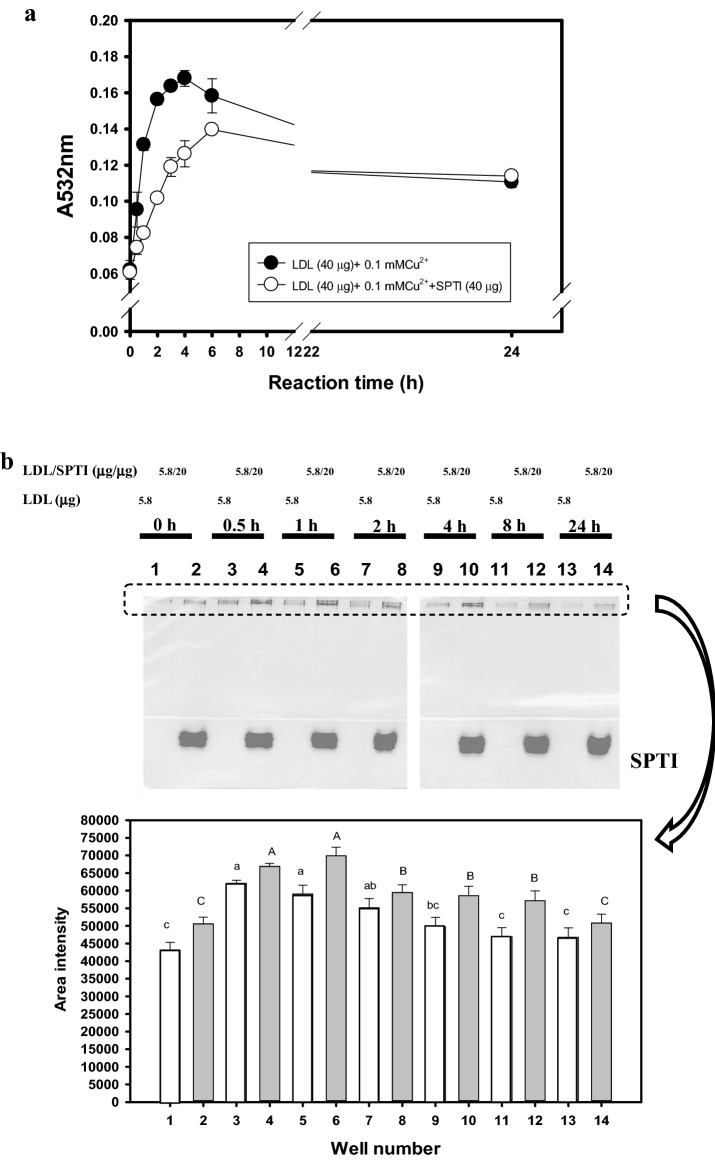
**a** Effect of sweet potato trypsin inhibitors (SPTIs) on 0.1 mM Cu^2+^-mediated low-density lipoprotein (LDL) oxidation during 24-h reactions evaluated by TBARS methods, and **b** during Cu^2+^-mediated LDL oxidation, effects of SPTIs on LDL or oxLDL binding were evaluated by protein stains in native PAGE gels with two layers of separation gels. The selected square frame was quantified by a Syngene G:bBOX imaging system (Syngene, UK). Values were presented as mean ± SD and were analyzed using one-way ANOVA, followed by a post hoc Tukey’s test for multiple comparisons. The different marked symbols (LDL only, lowercase; LDL/SPTIs model, uppercase) in each bar were significantly different (*P *< 0.05)

Figure 1a showed results of the Cu^2+^-mediated LDL oxidations during 24-h (expressed as TBARS formation, A532 nm) without or with SPTIs additions. It was found that the LDL oxidation (TBARS formation) was growing rapidly, and 4-h reaction reached to the top and then gradually decreased. However, the parallel experiment of LDL oxidations in the presence of SPTIs showed to reduce Cu^2+^-mediated LDL oxidations in the first 6-h intervals and then gradually closed to the control. Figure 1b showed the protein stains in the native PAGE gel of the fixed ratio of LDL/SPTIs (5.8 μg/20 μg, W/W) or LDL only (5.8 μg) at Cu^2+^-mediated LDL oxidations during 0, 0.5, 1, 2, 4, 8, and 24-h intervals, and each band intensity in the selected square frame was quantified. The Cu^2+^-mediated LDL oxidations (lanes 3, 5, 7, 9, 11, and 13) could significantly increase intensity of protein stains (*P* < 0.05) in the LDL position compared those in the non-oxidized LDL (zero time, lane 1). The 0.5-h to 2-h LDL oxidations reached the highest band intensities (lanes 3, 5, and 7), and then gradually decreased (lanes 9, 11, and 13, respectively for 4-h, 8-h, and 24-h), which showed no significant difference (*P* > 0.05) compared to that in the zero time (lane 1).

While, SPTIs additions in Cu^2+^-mediated LDL oxidations (lanes 4, 6, 8, 10, 12, and 14), the intensities of protein bands in the LDL position at each time interval of 0.5-, 1-, 2-, 4-, 8-, and 24-h reactions were enhanced compared to SPTIs additions without LDL oxidation (lane 2, zero time). It was noted that non-oxidized LDL in the presence of SPTIs (lane 2) showed higher intensity of protein stains in the LDL position (assumed as SPTIs/LDL complex) compared to that in the non-oxidized LDL only (lane 1), which showed SPTIs might bind to non-oxidized LDL to form SPTIs/LDL complex (lane 2). The 0.5-h and 1-h LDL oxidations in the presence of SPTIs reached the highest band intensity (lanes 4 and 6) and then gradually decreased (lanes 8, 10, 12, and 14, respectively for 2-h, 4-h, 8-h, and 24-h). The intensity of protein stains in 24-h reaction showed no significant difference (*P* > 0.05) compared to that in the zero time in the presence of SPTIs (lane 2), but showed higher intensities compared to the non-oxidized LDL only (lane 1).

### SPTIs bound to oxLDL revealed by native PAGE gels and HPLC

**Fig. 2:**
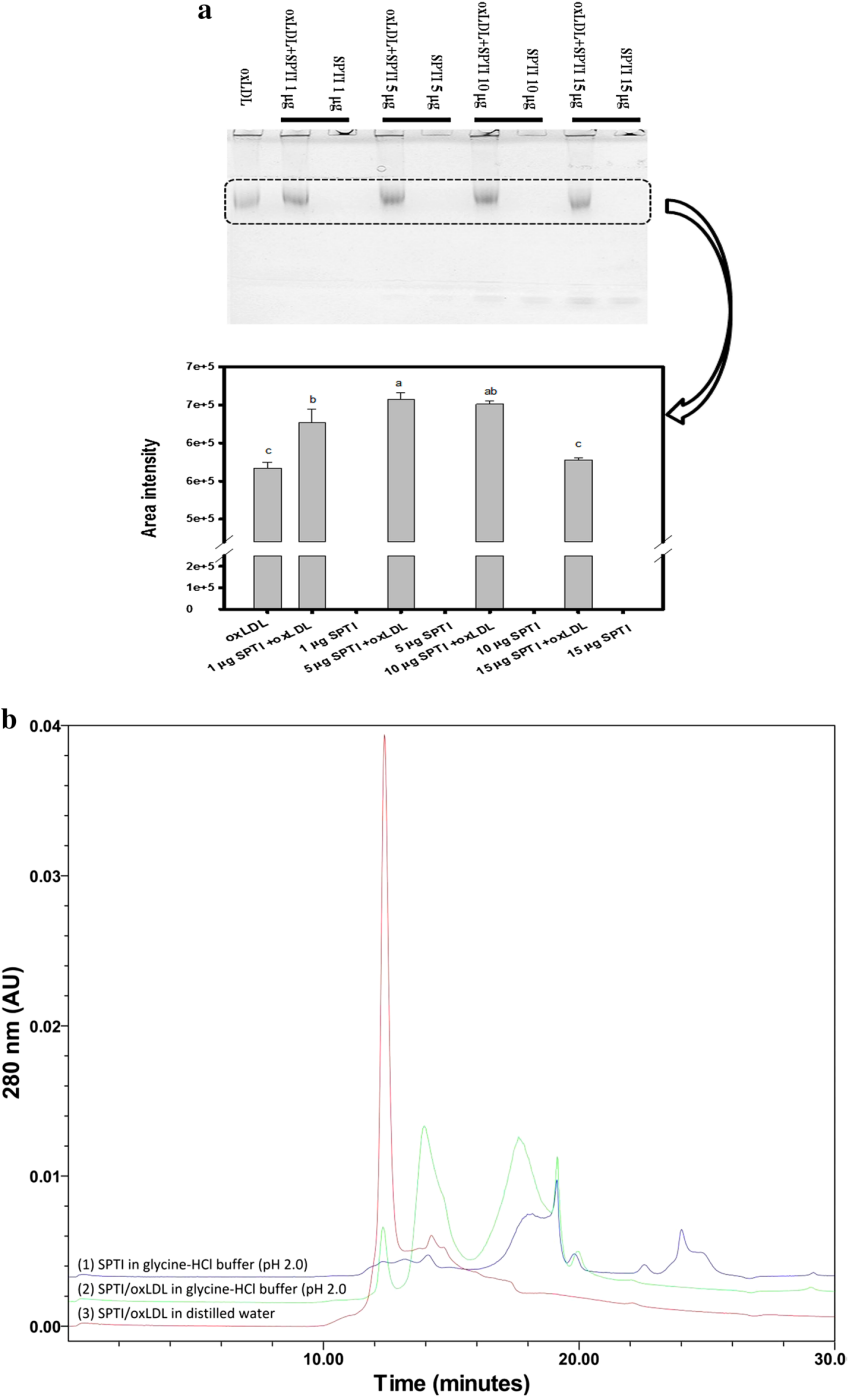
**a** Effects of SPTIs on oxLDL binding were evaluated by protein stains in native PAGE gels with two layers of separation gels. The selected square frame was quantified by a Syngene G:bBOX imaging system (Syngene, UK), and **b** the HPLC equipped with TSKgel G3000PW_XL_ (7.8 × 300 mm) gel filtration column was used to analyze the molecular size changes of SPTIs/oxLDL complex. (1) The SPTIs alone in glycine–HCl buffer (pH 2.0, deep blue line); (2) SPTIs/oxLDL binding assays in the glycine–HCl buffer (pH 2.0, green line); (3) SPTIs/oxLDL binding assays in the distilled water (red line). Values were presented as mean ± SD and were analyzed using one-way ANOVA, followed by a post hoc Tukey’s test for multiple comparisons. The different marked symbols in each bar were significantly different (*P *< 0.05)

The dialyzed LDL was reacted with copper (II) sulfate at 37 °C for 24 h, and the reaction was terminated by adding EDTA solution as the oxLDL. The SPTIs were mixed with oxLDL in tenfold-diluted PBS at 37 °C for 30 min and then performed native electrophoresis. Figure 2a showed the protein stains in the native PAGE gel of the different ratios of fixed amounts of oxLDL (10 μg) with different amounts of SPTIs (1, 5, 10, and 15 μg). The SPTIs only (1, 5, 10, and 15 μg) or LDL only (10 μg) were used as the controls, and the band intensity in the selected square frame was quantified. The addition of SPTIs at dose of 1, 5, 10, and 15 μg, seemed to enhance protein stains of oxLDL band, but only SPTIs addition at doses of 1, 5, and 10 μg showed significant differences compared to that of oxLDL only (*P* < 0.05).

The HPLC equipped with gel filtration column was used to analyze the molecular size changes of SPTIs bound oxLDL (Fig. 2b). There were two conditions for SPTIs/oxLDL binding assays, which the reaction was in the distilled water (red line of gel filtration chromatogram) or in the glycine–HCl buffer (pH 2.0) (green line of gel filtration chromatogram). The main peak of high molecular size of SPTIs/oxLDL complex was around 12 min in distilled water (red line). While in the glycine–HCl buffer (pH 2.0, green line), the amounts of original high molecular size (assumed as SPTIs/oxLDL complex) was reduced and shifted to smaller molecular sizes at 14 min and 18 min of HPLC chromatogram, which was similar to positions of SPTIs in glycine–HCl buffer (pH 2.0, purple line). Based on the results of Fig. 2, the SPTI could directly bind to oxLDL and low pH condition (pH 2.0) might break the interactions.

### LDL or oxLDL bound onto self-prepared SPTIs-Sepharose 4B affinity column

**Fig. 3:**
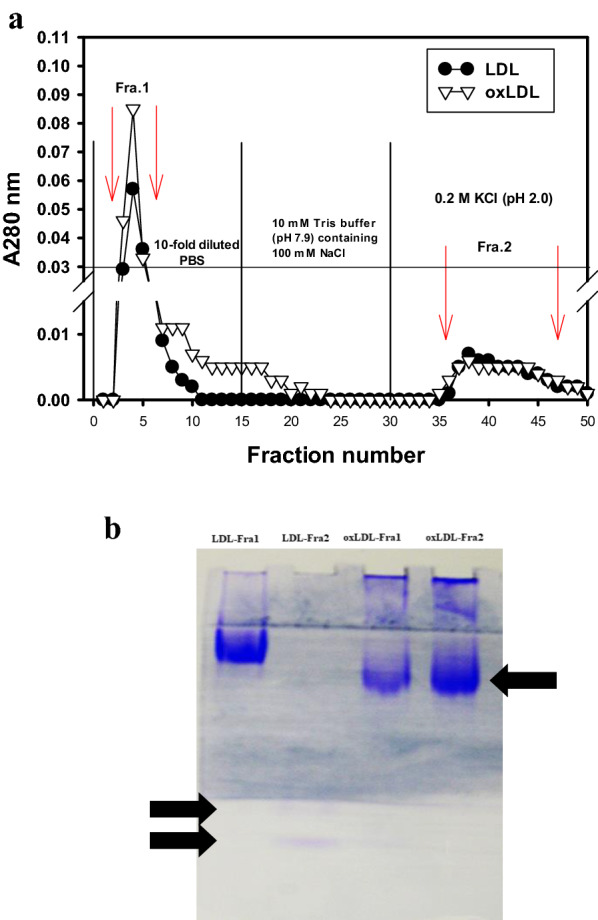
**a** Chromatograms of LDL or oxLDL on a SPTIs-Sepharose affinity column. Fraction 1 (Fra.1) was the unbound portion of LDL or oxLDL washed by tenfold-diluted PBS, and the fraction 2 (Fra.2) was the bound portion of LDL or oxLDL eluted by 0.2 M KCl (pH 2.0), and **b** the protein stains in the native PAGE gel with two layers of separation gels for LDL-Fra1, oxLDL-Fra1, LDL-Fra2, and oxLDL-Fra2. Arrows indicated the unidentified components from eluents

Figure 3a showed chromatogram of LDL or oxLDL on a SPTIs-Sepharose affinity column. Fraction 1 (Fra.1) was the unbound portion of LDL or oxLDL washed by tenfold-diluted PBS, and the fraction 2 (Fra.2) was the bound portion of LDL or oxLDL eluted by 0.2 M KCl (pH 2.0). It was found that some portions of LDL or oxLDL could be eluted from the SPTIs-affinity column by lowering pH to 2.0. Figure 3b showed the protein stains in the native PAGE gel of LDL-Fra1 and oxLDL-Fra1 or LDL-Fra2 and oxLDL-Fra2. The bound fraction (Fra. 2, arrow indicated) of LDL and oxLDL showed some different protein stains in the native PAGE gel. These results confirmed LDL or oxLDL could bind onto SPTIs, and interactions could be broken by lowering pH value to 2.0.

### Effects of native or reduced SPTIs and soybean TIs on LDL or oxLDL binding capacities

**Fig. 4:**
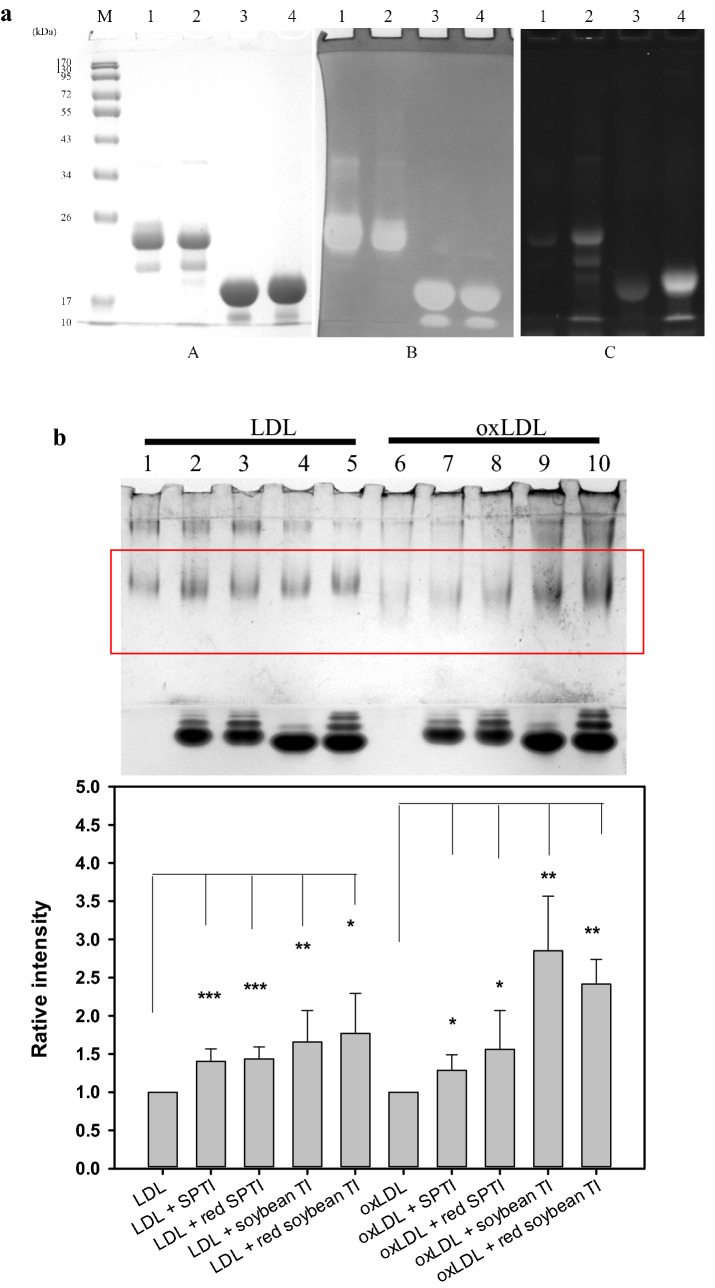
**a** Protein stains (panel A), activity stains (panel B), and free thiol-labeling stains (panel C). The lane 1 and lane 2, respectively, were native and reduced SPTIs; the lane 3 and lane 4, respectively, were native and reduced soybean TIs, and **b** effects of the native and reduced forms of SPTIs and soybean TIs on LDL and oxLDL binding capacities evaluated by the protein stains in the native PAGE gel with two layers of separation gels, and the selected square frame was quantified by a Syngene G:bBOX imaging system (Syngene, UK). Each of LDL, oxLDL, SPTIs, reduced SPTIs, soybean TIs, and reduced soybean TIs were 10 μg in the mixture. The lanes 1 to 5, respectively, were LDL only, LDL/SPTIs, LDL/reduced SPTIs, LDL/soybean TIs, and LDL/reduced soybean TIs. The lanes 6 to 10, respectively, were oxLDL only, oxLDL/SPTIs, oxLDL/reduced SPTIs, oxLDL/soybean TIs, and oxLDL/reduced soybean TIs. Data were expressed as mean ± SD. The student’s t-test was used for two group comparisons of binding capacities between LDL and (LDL + native or reduced forms of SPTIs or soybean TIs), or oxLDL and (oxLDL + native or reduced forms of SPTIs or soybean TIs). A difference was considered statistically significant when *P* < 0.05 (*) or *P* < 0.01 (**) or *P* < 0.001 (***)

Figure 4a showed the protein stains (panel A), activity stains (panel B), and stains of free thiol-labeling (panel C). The lane 1 and lane 2, respectively, were native and reduced SPTIs; the lane 3 and lane 4, respectively, were native and reduced soybean TIs. From the results of panel C of Fig. 4a, the reduced SPTIs (lane 2) and the reduced soybean TIs (lane 4) showed more intense thiol-labeling stains compared to each native proteins. The native or reduced forms of SPTIs and soybean TIs all showed trypsin inhibitory activities (panel B, Fig. 4a), and the main proteins of SPTIs (around 25 kDa) showed higher molecular size compared to that of soybean TIs (around 20 kDa) in the SDS-PAGE gels (panel A, Fig. 4a). These native and reduced forms of SPTIs and soybean TIs were used to test LDL and oxLDL binding capacities by the protein stains in the native PAGE gel (Fig. 4b), and the selected square frame was quantified by a Syngene G:bBOX imaging system (Syngene, UK). The lanes 1 to 5 (Fig. 4b), respectively, were LDL only, LDL/SPTI, LDL/reduced SPTI, LDL/soybean TI, and LDL/reduced soybean TI. The lanes 6 to 10 (Fig. 4b), respectively, were oxLDL only, oxLDL/SPTIs, oxLDL/reduced SPTIs, oxLDL/soybean TIs, and oxLDL/reduced soybean TIs. Under the same binding conditions, it was found that the native and reduced forms of SPTIs and soybean TIs showed LDL and oxLDL binding capacities and showed significant differences compared to the LDL or oxLDL only (*P* < 0.05, 0.01, 0.001). It was noted that the soybean TIs (native or reduced forms) showed higher average oxLDL binding capacities than those of SPTIs under the same amounts used (lanes 7 to 10) based on selected square frame quantifications. It was found that the soybean TIs (native or reduced forms) showed higher oxLDL binding capacities (lanes 9 and 10) than those of LDL (lanes 4 and 5) under the same amounts used. The native SPTIs showed similar binding capacities toward LDL and oxLDL (lanes 2 and 7), and reduced SPTIs also showed the similar results (lanes 3 and 8).

## Discussion

The present results showed that the SPTIs exhibited LDL binding capacities and oxLDL binding capacities, which might be related to lower TRARS levels during Cu^2+^-mediated LDL oxidations. The reduced SPTIs, soybean TI, and reduced soybean TI also showed LDL and oxLDL binding capacities. Though SPTIs (or sporamin) and soybean TI used in the present study were belonged to the Kunitz-type trypsin inhibitors, both amino acid sequences shared only 30% identity and 50% similarity (Senthilkumar and Yeh 2012). It was reported that the purified NADPH/thioredoxin system could facilitate SPTIs reductions in vitro and might beneficial as nitrogen sources for SP sprouting in vivo (Huang et al. 2004). The purified SPTIs showed thiol-disulfide interchanges to regenerate ascorbate from dehydroascorbate (as dehydroascorbate reductase activity), and the free thiol labeling stains of SPTIs were reported (Hou and Lin 1997a). Therefore, parts of purified SPTIs might exhibit free thiol stains in nature (Fig. 4a, lane 1 of panel C). It was reported SPTIs exhibited free radical scavenging activities, including DPPH radicals and hydroxyl radicals (Hou et al. 2001, 2005), and the scavenging activities were contributed by Cys residues for the former and by Trp residues for the latter (Hou et al. 2005). The higher concentrations of SPTIs (12, 15, and 18 mg/ml purified from TN57 SP cultivar; 4, 5, and 6 mg/ml from TN65 SP cultivar) were used significantly to reduce Cu^2+^-mediated LDL oxidation, which was catalyzed by 10 μM Cu^2+^ at 37 °C and determined by TBARS methods at the fixed 24-h reaction (Hou et al. 2005). The present study showed that no protective activity of SPTIs against LDL oxidation was found at fixed 24-h reaction, however, in the first 6-h SPTIs additions showed to reduce LDL oxidations and also formed LDL/SPTIs or oxLDL/SPTIs complex.

The chemical compositions of LDL particle comprised a glycoprotein of apoprotein B-100 (apoB-100), regulatory proteins, a monolayer of phospholipid, and a hydrophobic core, which totally accounted for about 75% total LDL weights (Alipov et al. 2017). The activated monocytes, macrophages, and endothelial cells in vivo could produce reactive oxygen species, lipoxygenase, and peroxidase, along with trace metal ions (Fe^3+^ and Cu^2+^) to enhance LDL oxidations. The processes of LDL oxidation generated fatty acid oxidation products, lipid-derived products and lipid-protein adducts, and protein cross-linking products via oxidations (Parthasarathy et al. 2010; Alipov et al. 2017). The 4-hydroxy-2-nonenal (HNE), one of reactive lipid products, was identified by LC/MS/MS as the lipid-protein adducts in histidine residue of the apoB-100 tryptic hydrolysates in Cu^2+^-mediated LDL oxidation (Bolgar et al. 1996). The oxLDL, but not LDL, could bind receptors to enhance oxLDL uptakes and lipid accumulations in subendothelial intima to form the foam cells and the advanced plaque, which the levels of oxLDL were used as a biomarker of endothelial dysfunction (Alipov et al. 2017).

The human LDL (or apoB-100) formed complex in vivo with endogenous oxidized α1-antitrypsin (oxidized AT) isolated from LDL fraction by the oxidized AT antibody-coupled affinity column, and LDL/oxidized AT complex was also found in atherosclerotic lesions and the arterial wall (Mashiba et al. 2001). The secretory AT, belonged to the serpin family to regulate serine proteinase, was produced mainly by liver or lipopolysaccharide-stimulated macrophages. The main function of AT was showed irreversibly to bind and inactivate neutrophil elastase or to inhibit excess proteinase activities in the inflammatory sites (Knoell et al. 1998). However, the oxidized AT lost the inhibitory activity against proteinases by oxidizing methionine residue to methionine sulfoxide in the active site by peroxide, peroxynitrite, or hydroxyl radicals attacks (Mashiba et al. 2001). The LDL and oxLDL could bind onto SPTIs-affinity column (Fig. 3) and some unidentified components were eluted by 0.2 M KCl–HCl buffer (pH 2.0). It was not clear the bound components from LDL or oxLDL onto SPTIs-affinity column. It will be possible to isolate and identify the eluents (or binding components) from the SPTIs-affinity column by LC/MS/MS techniques.

The Asp-hemolysin was a hemolytic toxin and produced by *Aspergillus fumigatus*, which was reported to form complex with oxLDL in Cu^2+^-mediated LDL oxidation during 24-h reactions, and 4-h Cu^2+^-mediated LDL oxidation showed the highest bindings (Kudo et al. 2001, 2002). The Asp-hemolysin also formed complex with LDL to the lesser extent (Kudo et al. 2001). The Asp-hemolysin showed to bind lysophosphatidylcholine (LysoPC) portion in oxLDL particles (Kudo et al. 2002). The synthesized peptides derived from Asp-hemolysin, such as IKNASLSWGKW**YKDG**DKDAEI (P-21, peptide contained 21 amino acids) and **YKDG** (P-4, peptide contained 4 amino acids), showed oxLDL and LysoPC binding capacities, which the YKDG moiety in Asp-hemolysin might be the important region for oxLDL or LysoPC bindings (Kumagai et al. 2006). Based on the present results, it will be possible to synthesize biotinylated-peptides derived from peptic hydrolysis of SPTIs to evaluate the LDL and oxLDL bindings to identify binding regions in SPTIs for LDL and/or oxLDL.

## Conclusion

In conclusion, native SPTIs showed to reduce copper ion-mediated LDL oxidations. The native or reduced forms of SPTIs and soybean TIs showed LDL binding and oxLDL binding capacities in vitro. The free Cys residues and Trp residues in SPTIs (or in reduced forms of SPTIs), respectively, showed to contribute DPPH radical and hydroxyl radical scavenging activities (Hou et al. 2005), which might scavenge lipid peroxyl radicals induced by Cu^2+^ to break lipid chain reactions of LDL oxidations. The SPTIs might contain regions that could bind to apoB regions in LDL or oxLDL, which the LDL/SPTIs or oxLDL/SPTIs mixtures will investigate by anti-apoB antibody in native gels. Later, the SPTIs or its synthesized peptides will intervene in hypercholesterolemia animal models to evaluate the effects on LDL levels, which will investigate in the future.

## Data Availability

All data generated during the study are interpreted in the manuscript.
